# Mitigating Physiological Responses to Layoff Threat: An Experimental Test of the Efficacy of Two Coping Interventions

**DOI:** 10.3390/ijerph13030338

**Published:** 2016-03-18

**Authors:** Tahira M. Probst, Lixin Jiang

**Affiliations:** 1 Department of Psychology, Washington State University Vancouver, 14204 NE Salmon Creek Ave., Vancouver, WA 98686, USA; 2 Department of Psychology, University of Wisconsin Oshkosh, 800 Algoma Blvd., Oshkosh, WI 54901, USA; jiangl@uwosh.edu

**Keywords:** emotion-focused coping, problem-focused coping, expressive writing, anticipated job loss, galvanic skin response

## Abstract

The purpose of the current study was to assess real-time physiological reactions to the threat of layoffs and to determine whether the use of an emotion-focused *vs.* problem-focused coping intervention would be more efficacious in attenuating these physiological reactions. A 2 (coping intervention) × 4 (within-subjects time points) mixed experimental design was used to test the hypotheses. Eighty-four undergraduates participated in this laboratory experiment during which their galvanic skin response (GSR) and heart rate (HR) were continuously monitored. Analyses indicate that individuals instructed to utilize an emotion-focused coping strategy experienced a significantly greater decline in their GSR compared to those utilizing the problem-focused coping method. Results suggest organizations conducting layoffs might focus first on dealing with the emotional aftermath of downsizing before focusing on problem-solving tasks, such as resume writing and other traditional outplacement activities.

## 1. Introduction

Even in the absence of a recession, organizations frequently downsize for a variety of reasons (e.g., in response to global competition, need to reduce costs, technology changes, *etc.*). In 2012 alone, there were 6500 extended mass layoff events across the United States, resulting in the layoff of more than 1.25 million workers [1]. Not surprisingly, nearly half of all Americans report being concerned about the future stability of their job [2]. Therefore, one of the most pressing issues for human resource professionals is to better understand not only the effects of such job insecurity concerns on employee health and well-being, but also how best to manage the inevitable career transitions when layoffs need to be implemented.

Over four decades of research suggest that the threat of job loss can have negative health consequences for employees. In one of the earliest studies, Cobb [3] found that the stress of possible job termination was associated with significant increases in norepinephrine excretion, serum creatinine, serum uric acid, and serum cholesterol. Research since then (e.g., [4,5,6,7,8]) has supported the finding that employees worried about job loss report a greater incidence of adverse physical health symptoms and conditions compared to secure employees. More recent longitudinal research (e.g., [9,10,11]) has also demonstrated that chronic job insecurity predicted changes over time in physical symptoms.

While research on the effects of job insecurity and threatened layoffs has consistently found significant negative effects on physical health outcomes, no research to date has yet collected real-time data to demonstrate the immediate physiological reactions of individuals who are notified of an impending layoff. This is understandable given the practical limitations of doing so in the context of an actual organizational layoff. In the current laboratory experiment, we measured in real-time the physiological reactions (galvanic skin response and heart rate) of research participants prior to receiving a layoff notice and during the reading of the notice. Galvanic skin response (GSR) and heart rate (HR) are commonly used in psychophysiology experiments to infer emotional state and cortical arousal in response to stressful situations. It was expected that GSR and HR would increase significantly when participants read the layoff notice.

The more central contribution of this study was to examine the effectiveness of two coping interventions at alleviating the stress response experienced by participants following the layoff notice. Such information can have practical applicability for human resource professionals as they assist their employees with difficult career transitions. In particular, there has been a significant amount of research examining the efficacy of problem-focused *vs.* emotion-focused coping at reducing the negative outcomes of unemployment. However, this research has been largely correlational taking an individual differences approach in the self-reported coping styles utilized by layoff victims, rather than using an intervention-based approach manipulating which coping strategy layoff victims are asked to utilize. While research indicates that a problem-focused approach may be better in regaining employment [12,13,14,15], it remains an empirical question as to whether an emotion- *vs.* problem-focused approach might be better in the *immediate* aftermath of a layoff at alleviating the victim’s stress response.

To explore the effectiveness of the emotion-focused coping and the problem-focused coping, participants in our study were instructed to either utilize an emotion-focused or problem-focused coping method during a five-minute writing period following the receipt of the layoff notice. It was expected that individuals utilizing the emotion-focused writing approach would experience a greater reduction in their stress-response compared to individuals instructed to use a problem-focused writing approach after receiving the layoff notice.

The following sections summarize the literature on the consequences of anticipated job loss, with the specific focus on physical health reactions of employees. Next, we utilize existing theory and research on problem-focused and emotion-focused coping methods to develop hypotheses regarding reactions to anticipated job loss.

### 1.1. Physical Health Consequences of Anticipated Job Loss

For the purpose of this study, anticipated job loss is defined as *advance notice of involuntary job loss due to layoffs*, rather than job loss due to being fired for cause [16]. Anticipated job loss differs from subjective job insecurity in a sense that the latter is regarded as *the perception that the future of one’s job is unstable or at risk*, regardless of any actual objective level of job security [16]. Unlike anticipated job loss, job insecurity is most commonly conceptualized as a subjective phenomenon that is best understood and described “in the eye of the beholder” (e.g., [17,18,19,20]), and is often measured by asking employees to indicate the perceived likelihood of losing one’s job. Upon receipt of an advance notice of layoffs, however, there is no ambiguity regarding one’s job security (*i.e.*, it is known that one’s job will end on a set date). Thus, while the stressor of impending job loss remains, the employee can begin to prepare and plan for re-entry into the job market [21].

Anticipated job loss also differs from job loss and unemployment. Job loss is a life event when paid employment is involuntarily removed from an individual (e.g., [22,23]), whereas unemployment is a state of being unemployed or not involved in a gainful occupation [22]. On the other hand, anticipated job loss is a state of being employed with the knowledge of impending job loss at a future date. Thus, anticipated job loss is differentiated from job loss and unemployment in that the affected individual remains employed for the time being.

Anticipated job loss can also be characterized as a transition process from being employed to being unemployed. Anticipated job loss leads to actual job loss, which, in turn, results in unemployment, unless an individual becomes immediately employed following job displacement. In this study, we will explore how individuals physiologically react to anticipated job loss and further explore how those reactions may differ as a function of the coping method employed in response to that job loss notification.

Lazarus and Folkman’s [24] transactional model of stress and coping provides a foundation for why threatened layoffs often lead to adverse outcomes. Stress occurs when individuals perceive a threat to their well-being or resources that exceeds their ability to cope with that event. Anticipated job loss inherently implies a potential threat to one’s well-being in the form of lost employment, income, and potentially-valued social status. As such, anticipated job loss is a stressor with expected detrimental physical reactions and other negative outcomes. Due to the unique nature of anticipated job loss (e.g., a short period of time) and the difficulties of data collection [25,26], studies on anticipated job loss are rare [27]. As such, we sought insights on this phenomenon from the job insecurity and unemployment literatures.

Past research has overwhelmingly documented adverse effects of both job insecurity (see [28] for meta-analyses, [29]) and unemployment (see [22,23] for reviews) ranging from negative cognitive, affective, and attitudinal reactions to reduced employee well-being and more negative employee behaviors. In our review below, we will mainly focus on the physical health outcomes of job insecurity and unemployment.

Job insecurity has negative physical health implications for employees on par with the health effects of a serious illness [9]. One of the earliest studies on this topic found that the stress of possible termination is associated with physiological responses such as increased norepinephrine excretion, serum creatinine levels, serum uric acid levels, and serum cholesterol levels [3]. More recent studies have shown that job insecurity is linked to decrements in general physical health [28,29], poor eating habits [30], high blood pressure, high cholesterol [31], and negative perceptions of good health across 16 European countries [32]. In a longitudinal study over extended periods of time ranging from three to 10 years, Burgard and colleagues [9] found that persistent job insecurity is consistently associated with significantly worse self-rated health than episodic or no job insecurity even after controlling for objective job loss over time, prior health, job characteristics, and socio-demographic characteristics.

Similar outcomes have also been documented in the unemployment literature. Unemployed individuals are reported to suffer from more headaches, stomachaches, sleep problems, lack of energy, and death from stroke, heart, and kidney disease than individuals who are employed [33]. Moreover, unemployment is related to an increase in disability, hypertension, ulcers, vision problems, cholesterol levels, and other doctor-diagnosed illnesses [22]. Recently, with two complementary, longitudinal population-based samples followed for periods of 15 to 35 years, Burgard, Brand, and House [9] found that even after controlling for numerous social background characteristics and baseline health, involuntary job loss is associated with significantly poorer overall self-rated health and more depressive symptoms.

The current laboratory experiment measured in real-time the GSR and HR of participants at multiple time points (e.g., prior, during, and after the receipt of the layoff notice). Based on the results from job insecurity [9] and unemployment literature (e.g., [9,34]), we proposed that participants would experience a significant increase in GSR and HR after reading the layoff notice.

*Hypothesis 1*: GSR and HR will significantly increase following receipt of a layoff notice (Time main effect).

### 1.2. Coping with a Layoff Notice: The Effect of Expressive Writing

Although we expected significant physiological responses to the layoff threat, we were primarily interested in examining the effectiveness of two coping interventions aimed at alleviating the stress response experienced by participants. As noted by Lazarus, stress occurs when a potential threat is perceived to exceed one’s ability to cope with or manage that threat [35]. Coping is about what people do in specific situations without reference to whether it works [36]. Coping has generally been classified into two broad categories: problem-focused coping and emotion-focused coping [37]. While problem-focused coping involves actually controlling and/or eliminating the cause of the actual problem, emotion-focused coping is aimed at improving the short-term well-being of the individual, primarily through decreasing the immediate feelings of distress [24]. In terms of coping with layoff notice, problem-focused coping strategies might include job search activities, retraining efforts, or geographic relocation to better job markets; in contrast, emotion-focused coping might include seeking out social support from friends and family, getting involved in community political action programs, and trying to block out thoughts about layoffs from one’s mind [38].

In general, researchers have theorized that problem-focused coping should be more closely related to job re-attainment and better psychological health than emotion-focused coping because problem-focused coping most directly reduces the source of the stressful situations [39]. The empirical evidence suggests, however, that the relationship between coping strategies and various outcomes is much more complex [40]. While some research (e.g., [12,13,15]), found that job search behaviors were associated with reemployment, other studies demonstrated that problem-focused coping did not facilitate reemployment (e.g., [38,41,42]).

In fact, Leana and Feldman [43] have argued that individuals who do not address the negative emotions associated with job loss, and who engage in job search too soon may not increase the opportunity of reemployment. Specifically, they suggested that if individuals do not address negative emotions following job loss, they may appear to be nervous and insecure in interviews and show low self-esteem, not to mention the increased possibility of making bad decisions about job search activities resulted from the trauma of the job loss. Conversely, individuals who are able to manage their negative emotions may improve the chances of receiving satisfied job offers by being stable and confident in interviews. This implies that individuals who try to use problem-focused coping too soon after a stressful event may actually prolong its negative consequences. Instead, a brief period of emotion-focused coping strategy might be suggested to precede problem-focused coping for problem-focused coping to be optimally successful [43].

It is also important to acknowledge that recent researchers have questioned the utility of a taxonomic system that distinguishes between emotion-focused *vs.* problem-focused coping [44,45]. We agree with previous researchers who note that these coping functions and strategies are likely complementary rather than competing in the face of a stressful situation [44]. Thus, individuals may spontaneously engage in more than one coping strategy and both may be functional in coping with the stressor at hand. While acknowledging this perspective, the purpose of the current study was to test the effectiveness of two coping interventions (one emotion-focused and one problem-focused) to evaluate which might be more effective in the *immediate* aftermath of receiving a layoff notice. In other words, while both may be functional, their utility may differ as a function of the timing in which they are utilized. In particular, we argue that the emotion-focused coping intervention (*i.e.*, expressive writing task) would be more effective than a problem-focused approach in the *immediate* aftermath of receiving a layoff notice. This hypothesis is particularly important to test, given that the focus of many outplacement activities often emphasizes a problem-solving approach (e.g., job search strategies, resume updating, *etc.*).

Expressive writing as an emotion-focused coping strategy has been shown to have a variety of benefits [46]. For example, respondents in an expressive writing condition showed a steady and significant decline in skin conductance levels [47,48], significant drops in physician visits from before to after writing (e.g., [49]), long-term improvements in mood and indicators of well-being (e.g., [50]), and lower subsequent absentee rate (e.g., [51]). Indeed, a recent meta-analysis confirmed that “expressive disclosure is effective, with a positive and significant average *r*-effect size of 0.075” [47] (p. 823).

Although the effects of expressive writing has been examined in various experimental settings and increasingly extended to the clinical setting (e.g., [52]), utilizing expressive writing to cope with stress in the workplace is still rare. An exception to this is a 1994 study conducted by Spera, Buhrfeind, and Pennebaker [53]. In their study conducted in an outplacement firm, 41 unemployed professionals were randomly assigned to either the expressive writing group or the writing control group, and the other 22 individuals were a non-writing control group. Participants in the expressive writing (writing about their deepest thoughts and feelings about the layoff) and writing control (writing about their activities in the job search) groups were required to write for five consecutive days, for 20 min each day. Eight months later, it was found that 52.6% of the expressive writing participants had found full-time employment, compared to 23.8% and 13.6% of writing control and non-writing control participants, respectively. Moreover, these outcomes were not related to the increased job search activities. In other words, participants in the expressive writing condition did not receive more phone calls, make more contacts, or send out more letters than controls. While their study sheds light on the important role of addressing the psychological issues of job loss in achieving the ultimate goal of reemployment, it only utilized self-report measures and did not capture any physiological results.

In the current laboratory experiment, we utilized the expressive writing paradigm to explore the effects of emotion-focused *vs.* problem-focused coping strategies in the face of impending job loss. Specifically, participants were told that they were going to be reading about an employee’s daily work activities, and they should try to imagine that they are in the same situation and consider how they might react to the various workplace situations for the effects of expressive writing on imaginary-trauma (see [54]). After receiving the layoff notice, participants were asked to write about either their emotional reactions to the layoff notice (*i.e.*, emotion-focused intervention) or to outline their next steps for their job search plan *i.e.*, problem-focused intervention; for the effects of a single writing session (see [55]). We expected that the emotion-focused writing group would show a greater drop in GSR and HR compared to that of the problem-focused writing group.

*Hypothesis 2*: GSR and HR will decrease to a greater extent among participants in the emotion-focused condition compared to the problem-solving condition (Time X Coping interaction).

## 2. Method

### 2.1. Pilot Study

In our laboratory experiment, we used an artificial scenario-based experiment where a layoff notice (*vs*. control condition) was manipulated. In an attempt to demonstrate the ecological validity of the scenario-based experiment, we conducted a pilot study to examine the effectiveness of the layoff manipulation before testing our hypotheses. If exposure to anticipated job loss would result in heightened job insecurity in the laboratory setting, this would provide support for ecological validity of the scenario-based experiment.

#### 2.1.1. Participants

Sixty undergraduates (*M* = 21.92 years, *SD* = 5.81) from a public university in the Midwestern United States participated in this pilot study in exchange for one point of extra credit. Of this group, 62% was female while 34% were male. 73% self-identified as Caucasian/White, while 25% self-identified as a racial/ethnic minority. 53% of the sample was employed at the time of the data collection, while 47% was not. 32% of the sample was holding or have had a supervisory role at work.

#### 2.1.2. Procedure

This pilot study was conducted using the online survey system Qualtrics (Qualtrics LLC, Provo, UT, USA). Once participants provided their informed consent, they were informed that they would be reading the background information about an employee and his daily work activities, and that they should try to imagine they are in the same situation and consider how they might react to the various workplace situations. The workplace scenario that followed described an employee named Kris Buford:

Kris Buford is a 45-year old man who works as a day-shift manager in a mid-sized food-processing company called PDX Industries. Kris has worked for PDX Industries for 15 years now working his way up to manager and has been pretty happy there. He receives full health care benefits, including medical, dental, and vision care, and is given three weeks of vacation each year, in addition to sick leave and a personal holiday.

Kris earns $45,000 per year, which is just enough for him and his family to get by. Kris and his wife have three children: one in college, one in high school, and just last year, an unexpected third child who is now six-months old. Kris’ wife quit her job to stay at home with the newborn, so they are dependent on his income to pay for the mortgage, tuition bills, groceries, car payments, and other necessities. They do not really have any family savings, which concerns Kris, but they have been able to get by.

Following the background information on Kris, participants read a summary of his daily activities as follows:

During a typical day, Kris arrives at the office at 8 am before the start of the day shift which begins at 8:30 am. He spends the half hour reading through and responding to company e-mails, and then makes his way out to the floor to greet his employees as they arrive. As one of the longest tenured employees in his company, Kris knows everyone by name and makes a point to inquire each day about his employees’ families, birthdays, and anniversaries, and other events that are going on in his employees’ lives. Although Kris is well-liked by his subordinates, he is not as close to upper-management at PDX Industries, but, he figures that is okay as long as they do not interfere with his job.

After that, participants were informed that the employee had just received an “Urgent Company Memo” in his Inbox. Participants were then randomly assigned to one of the two conditions (The randomization was implemented using the “randomizer” function in Qualtrics. We tested differences in demographics across two conditions to verify the effectiveness of randomization. There were no significant differences on the basis of: gender, age, race, employment status, part-time/full-time work status, managerial status, whether the participant had conducted layoffs in the past, or whether the participant him/herself had been laid off in the past). In the *layoff* condition, participants received notice that he was to be laid off from his/her organization. In the *control* condition, no mention of layoffs was given, but rather the memo concerned a flu outbreak among the employees and appropriate steps that should be taken to contain it. 

#### 2.1.3. Measures

Afterwards, participants were instructed to answer questions measuring their job insecurity levels. The *Job Security Satisfaction* scale [56]. Participants responded on a three-point scale (*yes, ?, no*) measuring the extent to which nine adjectives or phrases described their job security (e.g., “never been more secure”, “nerve-wracking”, “sufficient amount of security”). Responses were coded such that higher numbers reflected more job insecurity. The reliability of this measure (*i.e.*, Cronbach’s alpha) was 0.92.

Finally, a brief post-task questionnaire was administered to all participants to assess how easy or difficult it was for them to imagine that they were Kris in the scenario, and how concerned they were for Kris and his situation. All those questions were answered on a 1–7 Likert-type scale. Afterwards, all participants were debriefed.

#### 2.1.4. Pilot Test Results

As a manipulation check, an independent-samples *t*-test revealed that there was no significant difference between participants in the layoff condition and those in the control condition regarding how difficult to imagine themselves as Kris; overall participants reported it was relatively easy to imagine themselves in Kris’ place (*M* = 4.77, *SD* = 1.71). As expected, participants in the layoff condition (*M* = 5.90, *SD* = 1.19) expressed more concern for Kris than those in the control condition (*M* = 4.03, *SD* = 1.73), *t* (58) = 4.87, *p* < 0.001. Moreover, participants in the layoff condition reported a significantly higher level of job insecurity (*M* = 2.55, *SD* = 0.71) than those in the flu condition (*M* = 1.11, *SD* = 0.92), *t* (58) = 6.78, *p* < 0.001. Therefore, the results of the pilot study demonstrated the effectiveness of the layoff notice manipulation (Although we used a male character in the scenario, there were no significant gender differences on any of these measures). In other words, manipulating a layoff (*vs.* flu) notice might actually result in heightened job insecurity in the laboratory setting, thus providing support for ecological validity of the scenario-based experiment. Below we describe the physiological results from the second study as a function of the expressive writing task.

### 2.2. Laboratory Experiment

#### 2.2.1. Participants and Design

Eight-four undergraduates (*M* = 23.90 years, *SD* = 8.68) from a university located in the Pacific Northwest of the United States participated in this study. Fifty-seven of those who participated were female. Sixty-seven self-identified as Caucasian/White, while 17 self-identified as a racial/ethnic minority. 50% were currently employed (6% full-time). 30% currently or had held a supervisory position at work. Further, eight of the participants had conducted layoffs and 21 had been laid off in the past. Thus, those participants might easily relate to the described scenario.

Participants were randomly assigned to one of two conditions: the problem-focused coping writing condition (*N* = 42) or emotion-focused coping writing condition (*N* = 42) and their physiological responses were measured over four time periods (In order to extend the manipulation check results of the pilot test to our physiological outcomes of interest, a small number of additional participants (*N* = 14) were assigned to a control condition in which they were presented with a notice about a flu outbreak at work. The purpose of this control condition was to test the effectiveness of the artificial laboratory manipulation of a layoff notice at inducing physiologically-detectable levels of stress as measured by the GSR and HR, compared to the receipt of an alternative mildly stressful notice. Individuals exposed to layoff notices experienced a significantly greater increase in GSR compared to individuals in the flu notice control condition, *F* (1, 96) = 4.23, *p* < 0.05, η^2^ = 0.04, with GSR for those in the layoff condition increasing from 0.07 (*SD* = 0.13) to 0.36 (*SD* = 0.25), compared to 0.12 (*SD* = 0.05) to 0.23 (*SD* = 0.15) in the flu notice condition. However, this effect was not demonstrated for HR, *F* (1, 96) = 0.58, *ns*). Thus, this study employed a 2 (between-subjects expressive writing condition) × 4 (within-subjects timepoints) mixed experimental design.

#### 2.2.2. Procedure and Materials

Due to the monitoring equipment used, each participant was individually scheduled for the one-hour experiment. Upon entering the lab, all participants were provided with informed consent materials and given a brief orientation to the GSR and HR monitoring system and told that their participation would involve reading and writing about a workplace scenario. After signing the informed consent, participants were connected to a computerized system (Biopac BSL Psychophysiology and Neurophysiology system, Biopac Systems Inc., Goleta, CA, USA) that non-invasively measured physiological changes in GSR and HR for the duration of the one-hour study. The Biopac system uses sensors that attach to the skin with a temporary adhesive to monitor and record the autonomic nervous system (ANS) functioning (e.g., GSR and HR).

After being connected to the Biopac system, participants were given further instruction about the procedure of this experiment: first, they were asked to complete a demographic profile (*i.e.*, gender, age, race), after which they were given the hypothetical scenario to read. Although the Biopac system recorded the ANS through the whole study, we selected four time points to compare participants’ reactions: (1) while reading the background information of the hypothetical employee (a baseline reading); (2) while reading the Urgent Company Memo to the hypothetical employee containing the layoff manipulation (a notice reading); (3) while completing the coping writing intervention (a coping reading); and, (4) while completing their post-task questionnaire (a survey reading).

As in the pilot study, all participants read the background information about Kris and his daily work activities and were asked to imagine themselves in the same situation. Then all participants were informed of the Urgent Company Memo regarding the layoff notice.

Next, participants were randomly (As aforementioned, data were collected from one participant at a time, usually 2–3 participants each day over the course of a week. We randomized assignment condition in blocks of 14 switching back and forth (*i.e.*, 14 in the emotion-focused coping condition, 14 in the problem-focused coping condition, 14 in the emotion-focused coping condition, 14 in the problem-focused coping condition, *etc.*) until all 84 individuals were run) assigned to one of the two coping conditions and were told to begin their five-minute free-writing exercise. In the *emotion-focused coping* condition, participants were asked to write about their thoughts and emotions after receiving the layoff notice, *i.e.*, to express their feelings. Specifically, their instructions were as follows:

Imagining you were Kris and had just received a layoff notice, we would like you to take the next few minutes and write down what feelings you might have. In addition, describe how you might deal with the negative feelings often associated with unemployment (for example: isolation, anxiety, and depression).

In the *problem-focused coping* condition, participants were asked to think about how they might solve the problem of their impending job loss. Specifically, they were told:

Imagining you were Kris and had just received a layoff notice, we would like you to take the next few minutes and write down some ideas for how you might go about finding a new job. In other words, what specifically would you do to get a new job?

Following the free-writing exercise, a brief post-task questionnaire was administered to all participants to assess: (1) how realistic participants thought the scenario was; (2) how easy or difficult it was for them to imagine that they were Kris in the scenario; and (3) how concerned they were for Kris and his situation. All of those questions were answered on a 1–7 Likert-type scale. Following these questions, there was an open-ended question probing for any hypothesis guessing on the part of participants. Finally, all participants were debriefed.

### 2.3. Ethical Statement 

All subjects gave their informed consent for inclusion before they participated in the study. The study was conducted in accordance with the Declaration of Helsinki, and the protocol was approved by the Institutional Review Board of Washington State University (05901-004).

## 3. Results

### 3.1. Manipulation Checks

The open-ended question indicated that none of the participants guessed the purpose of the research. Preliminary analyses revealed that there was no significant difference between participants in the emotional-focused condition and those in the problem-focused condition regarding (1) how realistic the scenario was; (2) how difficult to image themselves as Kris; and (3) how concerned they were for Kris. Participants believed the scenario was quite realistic (*M* = 6.14, *SD* = 0.85), indicated that it was fairly easy to imagine themselves in Kris’ place (*M* = 5.20, *SD* = 1.45), and expressed relatively high concern for Kris (*M* = 5.88, *SD* = 1.00).

Analyses were also conducted to verify the effectiveness of randomization by testing for differences in demographics across the two conditions. There were no significant differences on the basis of gender, age, race, marital status, employment status, part-time/full-time work status, managerial status, or whether the participant had conducted layoffs in the past. The only variable where randomization was not completely successful was whether the participant him/herself had been laid off in the past, χ^2^ (1) = 5.14, *p* < 0.05, such that 35.7% of participants in the problem-solving condition had been laid off in the past, whereas only 14.3% of the expressive-writing participants had been. However, that variable was unrelated to our measures of GSR and did not interact with coping condition to predict changes in GSR during the study.

We also examined the content of writing samples provided during the expressive writing stage to verify that respondents followed the directions to provide emotion-focused *vs.* problem-focused narratives. Although these were not formally content-coded, sample emotion-focused statements included: “Feeling anger toward the company, worry and nervousness about how to break the news to my family” and “I feel very lost and confused. I am scared for what my family has to go through if I do not find another job”. Sample problem-focused statements included: “Use job search engine on the web; retraining myself and get a higher-level or related educational degree; find past bosses” and “Write a resume, get letters of recommendation. Search the internet job sites and go to local job services to find out who is hiring”.

### 3.2. Effects on GSR and HR

We first conducted a repeated-measures MANOVA including expressive writing condition (two), all time points (four), and both dependent variables (GSR and HR). The multivariate test of changes in GSR and HR over time was significant, F (6, 492) = 39.09, *p* < 0.001, η^2^ = 0.32. To specifically test Hypothesis 1, we conducted a planned comparison of GSR and HR at the two time points of reading the background information and receiving the urgent company memo. Using two univariate repeated-measures analyses, we found that individuals exposed to the layoff notice experienced a significant increase in their GSR (*F* (1, 83) = 69.43, *p* < 0.001, η^2^ = 0.46), and HR (*F* (1, 83) = 7.39, *p* < 0.01, η^2^ = 0.08). The GSR increased from 0.07 (*SD* = 0.13) to 0.36 (*SD* = 0.25) and the HR increased from 83.38 (*SD* = 13.45) to 84.89 (*SD* = 14.74). These results indicate that the layoff manipulation caused a significant increase in physiological GSR and HR. Thus, Hypothesis 1 was supported.

**Figure 1:**
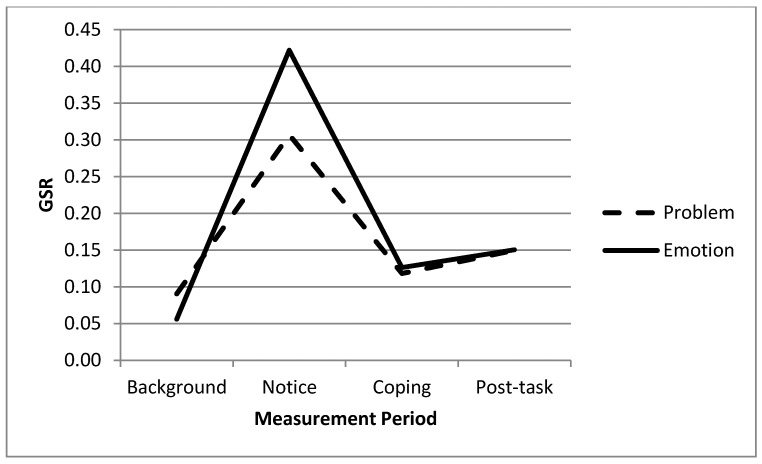
Coping method effects on GSR.

The multivariate test of changes in GSR and HR over time as a function expressive writing condition was also significant, F (6, 492) = 2.57, *p* < 0.05, η^2^ = 0.03. An inspection of the univariate effects, however, indicated that the expected Time x Writing interaction was only significant for GSR, F (3, 246) = 3.99, *p* < 0.05, η^2^ = 0.05. Thus, Hypothesis 2 was partly supported (similar to the pilot study, there was no gender difference in any analyses). Figure 1 below displays the form of the interaction. As can be seen, individuals in both groups began with similar GSR levels (emotion-focused mean = 0.06 and *SD* = 0.08; problem-focused mean = 0.09 and *SD* = 0.17). As noted above in our test of Hypothesis 1, GSR increased significantly for both groups. However, those instructed to utilize an emotion-focused coping strategy experienced a significantly greater decline in their GSR compared to those utilizing the problem-focused coping method. From reading the notice to writing the coping exercise, the GSR of participants in the emotion-focused group decreased from 0.42 (*SD* = 0.30) to 0.13 (*SD* = 0.03), while the participants’ GSR in the problem-focused group decreased from 0.31 (*SD* = 0.17) to 0.12 (*SD* = 0.04). Thus, while both groups experienced a decline in GSR, that decline was significantly greater in the emotion-focused writing condition. Both groups maintained low GSR levels (*M* = 0.15; *SD* = 0.03 in both groups) during the post-task survey completion phase.

## 4. Conclusions

The aim of the present study was to examine the real-time physiological responses to layoff threats and to determine the effectiveness of two possible coping strategies in a laboratory setting. Consistent with our predictions, we found that participants (even in an artificial laboratory setting) experienced an immediate measurable stress response in reaction to the layoff notice, as demonstrated by an increased level of GSR and HR. This result was consistent with the transactional theory of stress and coping [24] and past research documenting the negative health consequences of possible job termination [9] and unemployment [22].

Furthermore, those who were asked to write about their emotional reactions to the layoff notice experienced a significantly greater reduction in their stress response compared to those in the problem-focused condition. These results were consistent with the benefits of expressive writing [53] and confirmed Leana and Feldman’s [43] advice to address the emotional reactions to the layoff notice before taking further job search actions. Indeed, vocational counseling (e.g., [57]), job loss theory (e.g., [58]), and the process model for understanding victims’ responses to worksite/function closure [59] have argued that the grieving stages begin with the layoff notice and such grieving stages may be necessary for a layoff victim to go through in order to transition onto the next job. Despite the positive effects of such problem-focused coping as job search training on people’s mental health and reemployment (e.g., [60]), it may be more effective for human resource professionals to assist layoff victims with addressing their *emotional* reactions to the layoff notice *before* engaging in the job search activities. The results of our current study appear to provide empirical support for the importance of emotional coping, not only in the long term, but also in the immediate aftermath of receiving a layoff notice.

Although the GSR responses to the layoff notice and the writing exercise were as expected, HR did not appear to respond to the writing exercise. It may be that HR was not as sensitive as GSR to stressful situations such as the imaginary layoff notice among participants. Moreover, there was greater HR variability among the participants; as a result, while the effects were in the expected direction, there may not have been enough power to detect a significant effect.

### 4.1. Strengths and Implications

The majority of the literature on anticipated job loss, job insecurity, and unemployment has resulted from correlational, cross-sectional, and self-report data with few studies being able to establish the causal relationships between impending job loss and physical health consequences. Our laboratory study addressed this limitation of prior research by manipulating the experiences of the layoff notice and measuring the real-time physiological responses to the layoff notice. As predicted, we found increased levels of GSR and HR after the layoff notice, and confirmed the negative physiological effect of anticipated job loss on participants.

Similar to the job loss literature, the cross-sectional nature of research about coping with job loss precludes the assessment of causality. Moreover, the majority of research regarding coping with job loss has been conducted among participants who were looking for reemployment or have been reemployed. As such, this previous research was unable to demonstrate the efficacy of emotion-focused coping and problem-focused coping *immediately* following the layoffs notice. In our current study, in addition to manipulating the layoff experiences, we further manipulated the coping strategies used by participants. Participants were asked to either write about their emotional reactions to the layoff notice or their job search plan. Consistent with the expressive writing literature [54], we found that the emotion-focused writing decreased participants’ GSR to a larger extent than the problem-focused writing. This result might be able to explain the inconsistent findings regarding the effectiveness of emotion-focused coping and problem-focused coping strategies in the face of job loss because it seems that (at least, some) layoff victims might experience grieving stages and it is beneficial for victims to address their emotional reactions before engaging in job search activities.

These results have interesting implications for placement centers, employers, and employees faced with the threat of downsizing. Although we are not suggesting that organizations should offer the same five-minute writing task in the aftermath of a layoff notice, the empirical success of *focusing on emotions* (rather than problem solving) suggests that in the immediate aftermath of a layoff notice, placement centers and human resource managers should perhaps be more concerned with providing appropriate psychological counseling rather than with immediately focusing on outplacement assistance activities such as new job skills training or resume development. Moreover, such an emotion-focused intervention in the aftermath of a layoff notice might offset other forms of emotion-focused coping that have the potential to exacerbate the situation, e.g., harmful forms of emotional contagion from colleagues (see [61]).

### 4.2. Limitations and Directions for Future Research

Since this was a laboratory experiment, one could certainly critique the ecological validity of the study. The method used in this study was a scenario-based, role-playing study in which participants indicated how they would have responded to a situation described for them. Consequently, one could argue it is uncertain how real employees in a natural setting would react to actual layoff notices and whether the different coping mechanisms would be differentially effective in real situations. On the other hand, the manipulation checks performed in the pilot and main study did suggest that even in this artificial scenario-based experiment, there were pronounced effects on the research participants. Thus, one could also argue that true organization layoff victims might experience even more detrimental physiological responses to anticipated job loss, and derive more benefit from the emotion-focused writing. Indeed, Mook [62] argues that laboratory experiments are useful because they demonstrate the power of a phenomenon by showing it can happen even under artificial lab conditions.

Although we cannot claim that these results replicate a natural setting, they are noteworthy as the first known attempt to examine real-time physiological responses to layoff threats and coping strategies, something that would be exceedingly difficult to do within the context of an actual distribution of layoff notices. Nevertheless, constructive replications with other samples and research methods would certainly be advantageous in both confirming and further exploring the results that we found.

An additional limitation of the current study was the lack of a non-writing control group as a comparison to the emotion-focused writing and problem-focused writing conditions. It is possible that participants might naturally rebound to the original level of GSR without any intervention. Similarly, a problem-focused intervention may be arousing in and of itself because it inspires the worker to respond to the challenges of the layoff situation, whereas an emotion-focused coping intervention reduces arousal. To better tease out these effects, future research should add a third non-writing control group to compare the effects of emotion-focused and problem-focused writing with a non-writing group.

Although the participants’ GSR in the emotion-focused writing condition dropped to a greater extent than those in the problem-focused writing condition, the initial levels of GSR during the reading notice period among participants in the emotion-focused writing condition were higher than those in the problem-focused writing condition. This occurred despite the random assignment to conditions; nevertheless, of primary importance in the current study was that the slope of the decrease in GSR was significantly different between the two groups.

Finally, unlike traditional expressive writing tasks conducted over several days, our manipulation only involved one five-minute writing exercise. Future research might follow the traditional expressive writing procedure to explore the long-term effects of expressive writing on layoff victims’ physiological responses. However, we would argue that the current results are intriguing in that even with a brief intervention, significant effects were found between the expressive writing and job search conditions.

Despite the above limitations and the need for additional research to address those limitations, we believe that this study can serve as a useful starting point for better understanding employee responses to layoff threats and may have practical implications for downsizing organizations. Although prior research has found mixed results for the effectiveness of problem-focused *vs.* emotion-focused coping, both strategies are likely necessary and complementary for alleviating the negative consequences of being laid off; *cf.* [44,45]. Nevertheless, our results empirically support prior speculation that it may be more effective (from an acute physiological stress response perspective) to *immediately* deal with the emotional aftermath of a layoff notice before directing employees to problem-focused outplacement and job search services.
